# A case of hepatitis B virus reactivation after elranatamab therapy in a patient with multiple myeloma

**DOI:** 10.1186/s40780-025-00506-6

**Published:** 2025-10-23

**Authors:** Naoaki Nishimura, Hajime Nakashima, Kenji Yoshikuni, Akihiko Numata, Ryosuke Ogawa

**Affiliations:** 1 Department of Pharmacy, Japan Community Health Care Organization (JCHO) Kyushu Hospital, 1-8-1 Kishinoura, Yahatanishi-ku, Kitakyushu, Fukuoka 806–8501 Japan; 2 Department of Hematology and Oncology, Japan Community Health Care Organization (JCHO) Kyushu Hospital, 1-8-1 Kishinoura, Yahatanishi-ku, Kitakyushu, Fukuoka 806–8501 Japan

**Keywords:** Bispecific antibody, Elranatamab, Hepatitis B virus reactivation, Hypogammaglobulinemia, Intravenous immunoglobulin, Multiple myeloma

## Abstract

**Background:**

Multiple myeloma is an incurable hematologic malignancy. Although novel treatments have improved outcomes, many patients continue to relapse and eventually develop treatment resistance. Elranatamab, a bispecific antibody, has demonstrated promising efficacy in patients with relapsed/refractory multiple myeloma. However, clinical experience with elranatamab remains limited, and its immunomodulatory effects may increase the risk of opportunistic infections and viral reactivation. Here, we describe hepatitis B virus (HBV) reactivation in a patient with resolved HBV infection who was treated with elranatamab.

**Case presentation:**

A 76-year-old man with resolved HBV infection and treatment-resistant multiple myeloma received elranatamab. HBV DNA was not detected prior to treatment. On day 17 after initiation, an outpatient specimen returned as detectable but below the assay’s lower limit of quantification (< 1.0 log10 IU/mL). Elranatamab was discontinued due to hypotension, and the patient was subsequently hospitalized one week later with pneumonia. On day 78 after initiation, HBV DNA increased to 3.6 log10 IU/mL with transaminase elevation; tenofovir alafenamide 25 mg once daily was started the same day, after which HBV DNA declined to below the lower limit of quantification.

**Conclusions:**

Although elranatamab-specific information on HBV reactivation is limited, this case highlights a clinically relevant risk. Clinicians should remain vigilant for reactivation in patients receiving elranatamab, particularly those with resolved infection. Regular HBV DNA monitoring and prompt initiation of nucleos(t)ide analog therapy are essential to prevent severe complications, including fulminant hepatitis. Additionally, in the context of profound hypogammaglobulinemia during or after elranatamab, intravenous immunoglobulin may be considered to mitigate infection risk.

## Background

Multiple myeloma (MM) remains relapse-prone despite therapeutic advances, and outcomes are poor in heavily pretreated refractory disease [1, 2]. B-cell maturation antigen (BCMA) has emerged as a promising therapeutic target for MM [3–5]. Elranatamab is a BCMA–CD3 T-cell-engaging bispecific antibody (BsAb) that links BCMA on myeloma cells with CD3 on T cells, redirecting T-cell cytotoxicity toward myeloma cells and mediating antitumor effects [6, 7]. In the MagnetisMM-3 trial [6], elranatamab demonstrated a high response rate in relapsed/refractory multiple myeloma (RRMM), establishing it as a promising therapeutic option.

Patients with MM often exhibit reductions in immune cell populations, including CD4 + T cells, B cells, and natural killer cells. Chemotherapy further suppresses immunity, increasing the risk of opportunistic infections and viral reactivation [8, 9]. This T-cell engager (TCE) mechanism is also associated with impaired immune surveillance and hypogammaglobulinemia, increasing susceptibility to infections and viral reactivation [10–13]. Moreover, BCMA-targeted BsAbs affect normal plasma cells, which can prolong hypogammaglobulinemia and impair humoral immunity [11, 13–17]. Historically, infections—including hepatitis B virus (HBV)—have been major contributors to morbidity and mortality in MM [18].

Published clinical experience with BCMA-directed bispecific antibodies, including elranatamab, remains limited. While we identified no elranatamab-specific case reports of HBV reactivation, reactivation has been described with BCMA-targeted cellular or antibody therapies, suggesting a potential class effect and warranting close surveillance [7, 18]. HBV screening before chemotherapy is often suboptimal: in a 2016 U.S. report, Gonzalez et al. [19] found that fewer than 20% of patients in cancer centers were screened, whereas in a 2020 Japanese report, Yazaki et al. [20] reported a screening rate of 70.6%, though further improvement is needed. The Japanese HBV Treatment Guidelines (4th edition) recommend pre-chemotherapy screening and, for patients with resolved HBV, periodic HBV DNA monitoring (every 1–3 months) with on-demand nucleos(t)ide analog therapy [21]. In patients with resolved HBV receiving immunosuppressive therapy, HBV DNA monitoring with on-demand antivirals was more cost-effective than universal prophylaxis [22]; evidence derives from anti-CD20–treated B-cell non-Hodgkin lymphoma and is extrapolated to MM.

Here, we report a case of HBV reactivation after elranatamab administration in a patient with resolved HBV infection.

## Case presentation

HBV DNA values are reported as log10 IU/mL. Results below the assay’s lower limit of quantification (LLOQ, 1.0 log10 IU/mL [10 IU/mL]) are reported as below the LLOQ. Year X denotes the calendar year in which elranatamab was initiated.

A 76-year-old man with a history of resolved HBV infection was diagnosed with IgG-λ multiple myeloma in January, year X–11. Over the following years, he received BCD (bortezomib, cyclophosphamide, dexamethasone), DLd (daratumumab, lenalidomide, dexamethasone), and IsaPd (isatuximab, pomalidomide, dexamethasone) regimens, and underwent two nonconsecutive (non-tandem) autologous stem cell transplantations, each following high-dose melphalan. Despite these treatments, the disease relapsed and became refractory. Immediately prior to elranatamab, the patient was receiving IsaPd; the last dose was administered approximately five weeks before elranatamab initiation.

**Fig. 1:**
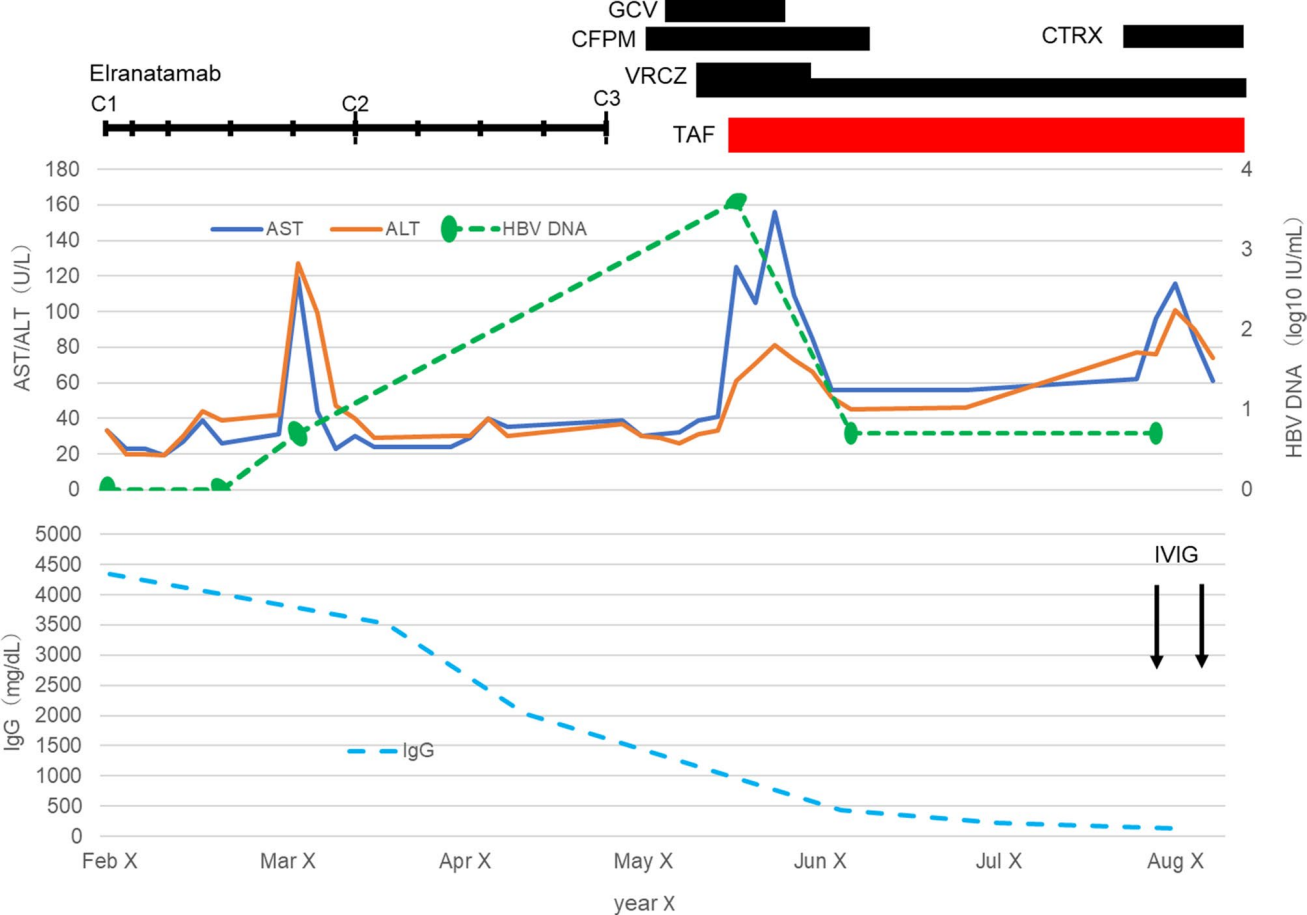
Clinical course of the patient with HBV reactivation after elranatamab therapy. Cefepime (CFPM) was started on admission for pneumonia, and ganciclovir (GCV) was initiated after cytomegalovirus (CMV) antigenemia was confirmed. The serum galactomannan (GM) index was positive (0.5); oral voriconazole (VRCZ) was started. HBV DNA was not detected in February. After the first cycle of elranatamab, HBV DNA became detectable but remained below the lower limit of quantification (LLOQ; <1.0 log10 IU/mL). On hospital day 17 (day 78 after elranatamab initiation), HBV DNA increased to 3.6 log10 IU/mL, accompanied by AST/ALT elevations to 125/61 U/L. Tenofovir alafenamide (TAF) was initiated on hospital day 17 (the same day as the HBV DNA surge and AST/ALT elevation), after which HBV DNA declined to below the LLOQ and remained below this threshold on subsequent measurements. During a subsequent admission, the patient developed pneumonia and received ceftriaxone (CTRX). Because of further hypogammaglobulinemia (IgG 128 mg/dL), intravenous immunoglobulin (IVIG) was administered. Note: HBV DNA values below the lower limit of quantification (LLOQ; 1.0 log10 IU/mL [10 IU/mL]) were plotted at 0.70 log10 IU/mL (LLOQ/2; 5 IU/mL). Not detected (negative) results were plotted at 0.00 log10 IU/mL (nominal value)

In February, year X, elranatamab therapy was initiated. Elranatamab was administered per the labeled step-up regimen in 28-day cycles: 12 mg on cycle 1 day 1 (C1D1) and 32 mg on C1D4, followed by 76 mg on C1D8 and then 76 mg once weekly thereafter (from C1D15 onward). HBV DNA was not detected prior to therapy. On day 17 after initiation, a specimen collected at an outpatient visit subsequently returned as detectable but below the LLOQ (< 1.0 log10 IU/mL). In April, elranatamab was discontinued for hypotension, and the patient was hospitalized one week later with pneumonia. During cycle 3, only C3D1 was administered; C3D8 was deferred and no further elranatamab was given. Ongoing medications included sulfamethoxazole/trimethoprim and valacyclovir for infection prophylaxis. The clinical course is summarized in Fig. 1, and selected laboratory data are shown in Table 1.

**Table 1:** Selected laboratory data on admission and during hospitalization

Parameter	Value	Unit
Complete blood count		
WBC	7600	/µL
Neutrophils	11	%
Lymphocytes	64	%
Monocytes	22	%
Hb	8.8	g/dL
PLT	9.4 × 10⁴	/µL
Biochemistry		
Total bilirubin	0.6	mg/dL
AST	39	U/L
ALT	37	U/L
ALP	115	U/L
GGT	121	U/L
LDH	324	U/L
BUN	18	mg/dL
Serum creatinine	1.69	mg/dL
Creatinine clearance	35.5	mL/min
Calcium	7.9	mg/dL
CRP	6.69	mg/dL
Immunoglobulin		
IgG	433	mg/dL
Serum free light chain		
κ-FLC	< 0.5	mg/L
λ-FLC	1.5	mg/L
κ/λ ratio	N.D. (κ < detection limit)	—
Hepatitis B virus serology		
HBsAg	Negative	—
HBsAb	1.2	mIU/mL
HBcAb (total)	Positive	—
HBV DNA	3.6	log10 IU/mL
Fungal markers		
Galactomannan (GM) index	0.5	—
(1,3)-β-D-glucan (BDG)	< 4	pg/mL

Results were obtained at admission unless otherwise noted. The following were measured during hospitalization: immunoglobulin G (IgG) and HBV DNA (3.6 log10 IU/mL on day 17 of hospitalization). Percentages may not sum to 100% due to atypical lymphocytes and minor leukocyte subsets. Serum galactomannan (GM) index ≥ 0.5 was considered positive (per institutional laboratory). Creatinine clearance was estimated using the Cockcroft–Gault equation

Abbreviations: ALP, alkaline phosphatase; ALT, alanine aminotransferase; AST, aspartate aminotransferase; BDG, (1,3)-β-D-glucan; BUN, blood urea nitrogen; CRP, C-reactive protein; GGT, gamma-glutamyl transferase; GM, galactomannan; Hb, hemoglobin; HBcAb, hepatitis B core antibody; HBsAb, hepatitis B surface antibody; HBsAg, hepatitis B surface antigen; HBV DNA, hepatitis B virus deoxyribonucleic acid; IgG, immunoglobulin G; κ-FLC, kappa free light chain; LDH, lactate dehydrogenase; λ-FLC, lambda free light chain; PLT, platelet count; WBC, white blood cell

On admission, empirical intravenous cefepime was started. Cytomegalovirus (CMV) antigenemia by the HRP-C7 method showed 40 positive cells per 50,000 leukocytes, and intravenous ganciclovir (GCV) was initiated. The serum galactomannan (GM) index was positive (0.5) on admission; oral voriconazole was started on hospital day 12. GCV was discontinued on hospital day 14 after CMV antigenemia resolved. On hospital day 17 (day 78 after initiation), HBV DNA increased to 3.6 log10 IU/mL, accompanied by elevated liver enzymes (AST/ALT 125/61 U/L). Tenofovir alafenamide (TAF) 25 mg once daily was initiated the same day, and liver enzymes gradually improved. Medication adherence was assessed, as clinically appropriate, through medication reconciliation and patient verbal confirmation on admission and on readmission. Two weeks before admission, immunoglobulin G (IgG) was 2037 mg/dL but had decreased to 433 mg/dL during hospitalization.

One month after starting TAF, HBV DNA was below the LLOQ, and liver enzymes remained stable. Two months after TAF initiation, the patient was readmitted with pneumonia. IgG had further declined to 128 mg/dL. Intravenous immunoglobulin (IVIG) was administered to prevent worsening infection and potential HBV reactivation. AST/ALT rose to 116/101 U/L; however, adherence to TAF was confirmed, and HBV DNA remained below the LLOQ, indicating no HBV reactivation. Liver dysfunction was attributed to pneumonia and gradually improved with clinical recovery.

## Discussion and conclusions

Recent reports indicate that HBV reactivation can occur with TCE therapy, and an ahead-of-print case described reactivation after sequential teclistamab and talquetamab [23]. Beyond TCEs, CAR T-cell therapy has also been associated with HBV reactivation [24]. Available reports suggest that when recognized early and treated promptly, biochemical abnormalities are often mild to moderate, consistent with the present case [23]. HBV screening and antiviral initiation were routine. We therefore focus on two case-specific interventions: early tenofovir alafenamide for rising HBV DNA with biochemical activity, and intravenous immunoglobulin for profound hypogammaglobulinemia during/after elranatamab.

Immunoglobulin replacement therapy (IgG-RT) is generally recommended for patients with IgG levels below 400 mg/dL [11, 13, 16, 17, 25].

In the MagnetisMM-3 trial [6], 75.5% of patients developed hypogammaglobulinemia (IgG < 400 mg/dL), and 43% received IgG-RT. In the present patient, IgG was 433 mg/dL—slightly above this conventional threshold—but Lancman et al. [15] reported increased infection risk at IgG ≤ 433 mg/dL. Thus, although not formally hypogammaglobulinemic, the patient may have been at increased risk of infection, including HBV reactivation. Upon readmission for pneumonia, IgG had declined to 128 mg/dL, prompting IgG-RT initiation.

Fu et al. [24] reported HBV reactivation in 14.3% of patients with chronic HBV and 6.98% of patients with resolved HBV infection receiving BCMA-targeted chimeric antigen receptor (CAR) T-cell therapy. Because both T-cell engagers and BCMA-directed CAR T-cell therapy redirect cytotoxic T cells and often deepen hypogammaglobulinemia, a similar risk of HBV reactivation with elranatamab is biologically plausible, consistent with emerging TCE-associated reports [23]. Fulminant hepatitis due to HBV reactivation can be fatal; Hwang et al. [26] reported 23% mortality from liver failure in affected patients.

In this case, the increase to 3.6 log10 IU/mL satisfied a pragmatic definition of HBV reactivation and justified immediate antiviral initiation. Even if HBV reactivation occurs, prompt initiation of nucleoside/nucleotide analogs can prevent a fulminant course [19–21, 24, 27]. Notably, the temporal association between the HBV DNA surge and prompt biochemical improvement after TAF—despite ongoing antimicrobials—supports HBV reactivation as the principal cause of liver injury rather than drug-induced hepatotoxicity or infection-related transaminitis.

In this case, regular HBV monitoring was performed, and HBV DNA was not detected before initiating elranatamab. Prompt initiation of TAF upon HBV reactivation effectively reduced viral load and prevented fulminant hepatitis. Continued adherence monitoring helped prevent further reactivation. Elranatamab may pose a risk of HBV reactivation in patients with resolved infection; vigilant HBV DNA monitoring and early antiviral therapy are essential. Accordingly, because of profound hypogammaglobulinemia during/after elranatamab, we administered IVIG to mitigate infection risk. In our management, early TAF and IVIG were the principal interventions in this case.

## Data Availability

All data generated or analyzed during this study are included in this published article.

## Abbreviations

ALT: Alanine aminotransferase
AST: Aspartate aminotransferase
BCMA: B-cell maturation antigen
BsAb: Bispecific antibody
CAR T-cell: Chimeric antigen receptor T cell
CMV: Cytomegalovirus
GCV: Ganciclovir
GM: Galactomannan
HBV: Hepatitis B virus
HBV DNA: Hepatitis B virus deoxyribonucleic acid
IgG: Immunoglobulin G
IgG-RT: Immunoglobulin replacement therapy
IVIG: Intravenous immunoglobulin
LLOQ: Lower limit of quantification
MM: Multiple myeloma
RRMM: Relapsed/refractory multiple myeloma
TAF: Tenofovir alafenamide
TCE: T-cell engager
