# Publisher Correction: A case of hepatitis B virus reactivation after elranatamab therapy in a patient with multiple myeloma

**DOI:** 10.1186/s40780-025-00520-8

**Published:** 2025-12-09

**Authors:** Naoaki Nishimura, Hajime Nakashima, Kenji Yoshikuni, Akihiko Numata, Ryosuke Ogawa

**Affiliations:** 1 Department of Pharmacy, Japan Community Health Care Organization (JCHO) Kyushu Hospital, 1-8-1 Kishinoura, Yahatanishi-ku, Kitakyushu, Fukuoka, 806–8501 Japan; 2 Department of Hematology and Oncology, Japan Community Health Care Organization (JCHO) Kyushu Hospital, 1-8-1 Kishinoura, Yahatanishiku, Kitakyushu, Fukuoka, 806–8501 Japan

**Correction to: Journal of Pharmaceutical Health Care and Sciences (2025) 11:91.**

10.1186/s40780-025-00506-6.

The original publication of this article contained several errors related to the citation numbering which occurred during the publication process. The incorrect and correct information is shown in this correction article. The publisher apologizes to the readers & authors for the inconvenience.

Incorrect

- IgG levels below 400 mg/dL [11, 13, 16, 17, 24].
- Fu et al. [25]

Correct

- IgG levels below 400 mg/dL [11, 13, 16, 17, 25].
- Fu et al. [24].

